# Important applications of DNA nanotechnology combined with CRISPR/Cas systems in biotechnology†

**DOI:** 10.1039/d4ra08325c

**Published:** 2025-02-25

**Authors:** Yuqi Huang, Zhongping Chen, Huacui Huang, Shijia Ding, Mingjun Zhang

**Affiliations:** a Clinical Laboratory, Chongqing Jiulongpo District People's Hospital Chongqing 400050 China lijiankezhang@126.com; b Clinical Laboratory, Chengdu Xindu District People's Hospital Sichuan 610599 China; c Key Laboratory of Clinical Laboratory Diagnostics (Ministry of Education), College of Laboratory Medicine, Chongqing Medical University Chongqing 400016 China dingshijia@163.com

## Abstract

DNA nanotechnology leverages the specificity of Watson–Crick base pairing and the inherent attributes of DNA, enabling the exploitation of molecular characteristics, notably self-assembly, in nucleic acids to fabricate novel, controllable nanoscale structures and mechanisms. In the emerging field of DNA nanotechnology, DNA is not only a genetic material, but also a versatile multifunctional polymer, comprising deoxyribonucleotides, and facilitating the construction of precisely dimensioned and precise shaped two-dimensional (2D) and three-dimensional (3D) nanostructures. DNA molecules act as carriers of biological information, with notable advancements in bioimaging, biosensing, showing the profound impact. Clustered regularly interspaced short palindromic repeats (CRISPR) and CRISPR-associated systems (Cas) constitute self-defense mechanisms employed by bacteria and archaea to defend against viral invasion. With the discovery and modification of various functional Cas proteins, coupled with the identification of increasingly designable and programmable CRISPR RNAs (crRNAs), the potential of the CRISPR/Cas system in the field of molecular diagnostics is steadily being realized. Structural DNA nanotechnology provides a customizable and modular platform for accurate positioning of nanoscopic materials, for *e.g.*, biomedical uses. This addressability has just recently been applied in conjunction with the newly developed gene engineering tools to enable impactful, programmable nanotechnological applications. As of yet, self-assembled DNA nanostructures have been mainly employed to enhance and direct the delivery of CRISPR/Cas, but lately the groundwork has also been laid out for other intriguing and complex functions. These recent advances will be described in this perspective. This review explores biosensing detection methods that combine DNA nanotechnology with CRISPR/Cas systems. These techniques are used in biosensors to detect small molecules such as DNA, RNA, and *etc.* The combination of 2D and 3D DNA nanostructures with the CRISPR/Cas system holds significant value and great development prospects in the detection of important biomarkers, gene editing, and other biological applications in fields like biosensing.

## Abstract 2

Schematic illustration of several synthetic forms of 2D and 3D DNA nanotechnology combined with CRISPR/Cas systems in biotechnology.

## Graphical abstract

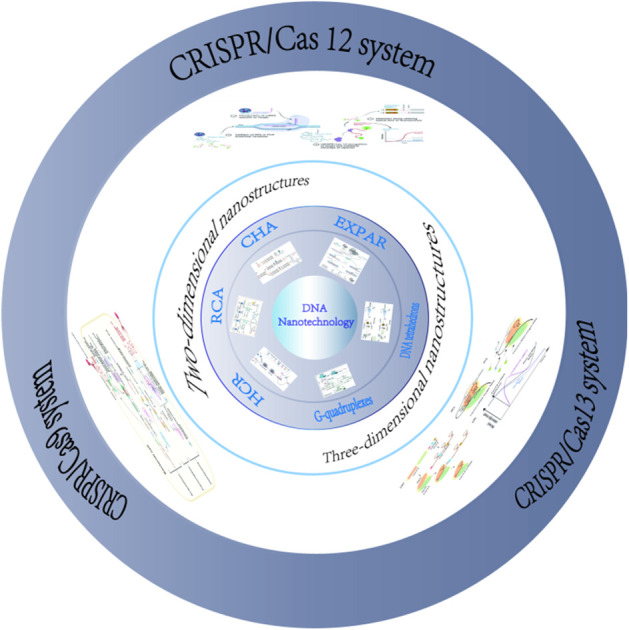

## 1. Introduction

DNA nanotechnology leverages the specificity of Watson–Crick base pairing and the unique properties of DNA, employing self-assembly strategies to construct stable and programmable two-dimensional (2D), three-dimensional (3D), and even multidimensional DNA nanostructures. These structures can integrate with other technologies to detect and deliver small molecules in biosensing applications, representing an innovative molecular nanotechnology. In the 1980s, Seeman first proposed the concept of using nucleic acids to construct self-assembled nanomaterials, which spurred the rapid development of DNA nanotechnology in the biological field. At this point, DNA was no longer seen merely as genetic material.^1^ Due to the inherent structural characteristics of DNA molecules, such as predictable intermolecular interactions (Watson–Crick base pairing), automated chemical synthesis, and enzymatic modification, many DNA or DNA-based nanostructures have achieved precise control of size and shape, as well as controllable self-assembly of surface chemistry and dynamic functions, in one-dimensional, two-dimensional, or three-dimensional forms.^2^ DNA nanostructures possess unique characteristics that endow them with powerful signal output capabilities.^3^ DNA, composed of deoxyribonucleotides, is a macromolecular polymer with good stability and programmability. It has found extensive utility as a versatile biomaterial to construct various nanostructures with precise dimensions and specific shapes. DNA nanotechnology represents an interdisciplinary research field that leverages the nanoscale dimensions, rigid structure, and robust encoding capabilities of DNA (deoxyribonucleic acid) to fabricate diverse nanostructures. These structures find applications across biomedicine, chemistry, and materials science, biomedicine, chemistry, and materials science, driving significant advancements in bioimaging, biosensing, and drug delivery. The evolution of DNA nanotechnology has revolutionized our perception of DNA from solely a genetic information carrier to a pivotal tool for engineering nanostructures.^4,5^

Clustered regularly interspaced short palindromic repeats (CRISPR) and CRISPR-associated systems (Cas) are self-defense mechanisms used by bacteria and archaea to resist viral invasion.^6^ They ensure specificity through the base pairing principle of guide RNA with target nucleic acids and achieve gene cutting and modification with the help of Cas proteins.^7^ The combination of CRISPR and its associated proteins (Cas) forms a powerful, efficient, and programmable molecular scissor capable of inducing genetic cuts at specified target sites. CRISPR/Cas effector proteins are diverse, varying in size, structure, composition, and targets.^8^ CRISPR/Cas, originally a part of microorganisms' adaptive immune system against viral pathogens, has undergone redesign to achieve precise nucleic acid recognition and subsequent cleavage. These advancements mark a new era of genetic manipulation. The CRISPR system, a prokaryotic adaptive immune system, enabling modulation of DNA replication, activation, transcription, and translation processes,^9^ has emerged as a revolutionary tool for genome editing, transcription regulation, and molecular diagnostics.^10^

In the past decade, CRISPR-Cas gene editing technology has revolutionized molecular biology experiments and instilled tremendous promise for the treatment of human genetic diseases. The CRISPR system is currently classified into two major classes with six types. The primary characteristic of the first class is the use of multiple Cas proteins working together to exert effects, including type I, type III, and type IV. The second class requires only a single effector protein to perform the functions of the multi-protein systems of the first class, making it the preferred choice for applications such as gene editing. This class is mainly divided into three types: type II, type V, and type VI,^11^ with corresponding effector proteins Cas9, Cas12, and Cas13, respectively. While most Cas9 and Cas12 variants exhibit DNA endonuclease activity, Cas13 variants demonstrate a distinct preference for RNA targeting and cleavage.^12^

**Scheme 1:**
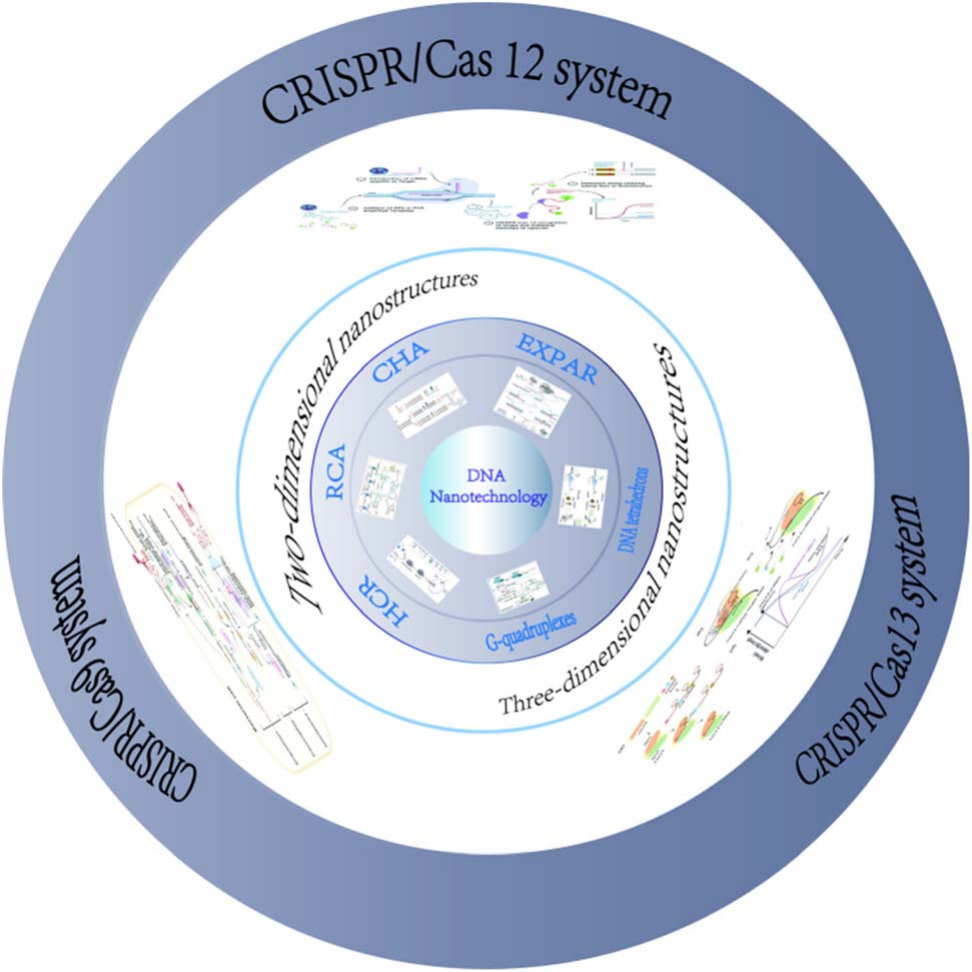
Schematic illustration of several synthetic forms of 2D and 3D DNA nanotechnology combined with CRISPR/Cas systems in biotechnology.

The discovery and refinement of various functional Cas proteins, coupled with the designable and programmable clustered regularly interspaced short palindromic repeats ribonucleic acids (CRISPR RNA, crRNA), have progressively unlocked the potential of the CRISPR/Cas system in molecular diagnostics. Recent years have witnessed burgeoning interest in its applications in biosensing and bioimaging, which are now prominent areas of exploration. Due to its precise nucleic acid targeting and cleavage functions, the CRISPR/Cas system can easily integrate with various signal amplification technologies, thereby enhancing its efficacy in biosensing applications. The combined application of these technologies in biosensing systems demonstrates high sensitivity and specificity,^13,14^ enabling the detection of ions, small molecules, proteins, enzymes, exosomes, pathogenic bacteria, viruses, and more, facilitating various signal output modes (Table 1). This review will focus on the classification and mechanism of CRISPR/Cas systems, as well as the 2D and 3D DNA nanostructures assembled with CRISPR/Cas systems and their significant roles in the field of biology,^48^ as shown in Scheme 1. It summarizes recent research progress, analyzes challenges faced in practical applications, and anticipates future trends in the research and application of DNA nanotechnology combined with the CRISPR/Cas system in biological detection, suggesting potential new research areas.^49,50^

**Table 1:** Biosensing application of DNA nanotechnology combined with CRISPR/Cas systems

CRISPR/Cas system	DNA nanostructure	Sensing mode	Biosensing application	Linear range	LOD	Ref.
CRISPR/Cas9 system	2D-DNA nanostructure	Cascade-HCR	The delivery of Cas9 RNP	—	—	15
		HCR	The detection of M.TB(H37Ra)	10^2^ to 10^8^ CFU mL^−1^	30 CFU mL^−1^	16
		CASLFA	The detection of Lm, GMO and ASFV	—	—	17
		CHA	MiR-21 detection and miR-21 imaging	1 pM to 10 nM	23.5 fM	18
		CHA	ASPs	—	—	19
		RCA and CHA	The detection of miRNA	10 fM to 100 pM	3.45 fM	20
		RACE	The detection of miRNAs	1 pM to 10 nM	90 fM	21
		RCA	The detection of *S. aureus*	10 CFU mL^−1^ to 10^7^ CFU mL^−1^	7 CFU mL^−1^	22
		RCA	The detection of miRNA let-7-5p	0.1 fM to 0.1 μM	10 aM	23
		CAS-EXPAR	The detection of ss-DNA	1 amoL to 10 fmoL	0.82 amoL	24
		CRISPR-EXPAR	The detection of target mutation	1 fM to 1 nM	437 aM	25
		ESDR	The detection of ctDNA	0.01 pM to 500 pM	0.13 pM	26
		CRISDA	The detection of target DNA	—	2.5 aM	27
		SDA-RCA	The detection of *E. coli* O157:H7	5 fM to 5 pM	1.87 fM	28
	3D-DNA nanostructure	GqRNA	Photo-reversible modality	—	—	29
		G-quadruplex motif	Improving genome editing	—	—	30
		Cas-G4EX	The detection of ssRNA	250 aM to 2.5 nM	250 aM	31
			The detection of ssDNA	100 aM to 1 nM	100 aM	
		G4/hemin	Synergistic PDT	—	—	32
		DNA-crosslinked nanogel	Target genome editing	—	—	33
CRISPR/Cas12 system	2D-DNA nanostructure	CRISPR-CHLFA	The detection of SASR-CoV-2	—	—	34
			The detection of MTB	—	—	
		CLAP	The detection of SARS-CoV-2	—	4 copies μL^−1^	35
		CHA	The detection of PCBs	500 fg mL^−1^ to 50 ng mL^−1^	128 fg mL^−1^	36
		RCA	The detection of dsDNA	40 pM to 90 pM	45.0 pM	37
		RCA	The detection of *Alternaria*	20 pM to 500 pM	22 pM	38
		RCA	The detection of FEN1	1 × 10^−6^ to 1 × 10^−3^ U μL^−1^	4.1 × 10^−7^ U μL^−1^	39
		cPER	The detection of RNase H	0.005 to 0.1 U mL^−1^	0.00061 U mL^−1^	40
		SRPL-TraCs	The detection of HIV-1	1 pM to 100 pM	0.13 pM	41
	3D-DNA nanostructure	DNA tetrahedron	The detection of HPV18	0.5 nM to 10 nM	3 nM	42
		G-quadruplex	The detection of *L. donovani*	5 pM to 250 pM	5 pM	43
		G-quadruplex	The detection of *S. enterica*	—	20 CFU	44
		HMPs	The detection of HPV	—	2 aM	45
CRISPR/Cas13 system	2D-DNA nanostructure	Enzymatic cycle amplification	The detection of miRNA	100 fM to 1 nM	83.2 fM	46
		RCA and PCR	The detection of cccDNA	—	—	47

## 2. DNA nanotechnology

The field of DNA nanotechnology was pioneered by Nadrian C. Seeman and his colleagues in the early 1980s. Based on the classical Watson–Crick base pairing rules and the self-assembly of DNA's basic components, predictable structures capable of producing a wide array of complex architectures were created.^51^ In particular, the integration of biotechnology and nanotechnology has led to the development of innovative technologies such as biosensors, drug delivery, and biological imaging.^52^ Nucleic acids are regarded as among the most crucial molecules in organisms and are hypothesized to have played a fundamental role in the origin of life on Earth. Since 2010, DNA nanotechnology has made significant strides, undoubtedly overcoming the limitations of DNA solely as genetic material. Therefore, DNA nanotechnology represents a novel approach for constructing biosensors, where nucleic acids offer significant advantages as biomolecular probes.^53^

DNA nanostructures are recognized for their exceptional editability and biocompatibility^54^^.^ The design and assembly of DNA nanstructures is a critical aspect of DNA nanotechnology. DNA nanstructures design typically relies on the high programmability and self-assembly capability of DNA molecules. Pairing complementary DNA fragments allow for the rational design and construction of nanstructures with predefined topologies and geometries.^55^ Leveraging the programmability of Watson–Crick base pairing and the binding capabilities of DNA with other molecules, artificially constructed DNA nanstructures have increasingly found complex and diverse applications in biosensors.^56^ DNA nanstructures exhibit precise geometric shapes, spatial addressing capabilities, and enhanced biological stability. DNA nanotechnology, with its programmability and self-assembly capability, has facilitated the construction of a variety of nanoscale devices and materials, spanning from simple 2D patterns to intricate 3D structures, thereby paving the way for new avenues in scientific and technological exploration.^57,58^ In biosensing applications, DNA nanstructures capitalize on their high molecular recognition, signal amplification, and transduction capabilities, providing new pathways for ultra-sensitive and highly selective biomolecule detection, promising significant advancements in future biosensing technologies.

## 3. CRISPR/Cas systems

The CRISPR/Cas system is an adaptive immune system discovered in bacteria and archaea, primarily employed to defend against viral and exogenous DNA invasion.^59,60^ This system consists of two main parts: CRISPR (Clustered Regularly Interspaced Short Palindromic Repeats) and Cas genes (CRISPR-associated genes), with the latter encoding CRISPR-associated proteins (Cas proteins).^61,62^ CRISPR/Cas systems are gene editing technologies originating from bacteria and archaea, enabling precise modification of specific DNA sequences. Initially, this system served as a natural immune mechanism in these microorganisms to defend against viruses attacks (such as phages). CRISPR/Cas systems are found in various bacteria and archaea, each with different compositions and mechanisms of action. According to evolutionary relationships, the CRISPR/Cas system can be classified into two major classes and six minor classes. The first class consists of multi-effector protein complexes, including types I, III, and IV, which are less frequently employed. The second class comprises single-effector proteins, including type II (Cas9 endonuclease), type V (Cas12 and Cas14 nucleases), and type VI (Cas13 nucleases).^63,64^ Due to their efficiency and simplicity, second class systems have found extensive applications in genome editing, gene expression regulation, and nucleic acid detection. The CRISPR/Cas system's ability to specifically recognize target sequences provides unique advantages for nucleic acid detection, particularly in the specific recognition of target DNA by Cas9 and Cas12a, and target RNA by Cas13a.^65^ Cas9, Cas12a, and Cas13a are the most studied and widely applied effector proteins of the CRISPR/Cas system. CRISPR consists of repetitive sequences—short, palindromic DNA sequences that repeat and spacer sequences—fragments extracted from foreign DNA (*e.g.*, viruses or plasmids), inserted between repetitive sequences to record and remember genetic information of invaders. CRISPR-associated proteins (Cas) associated with CRISPR sequences, responsible for executing various functions, particularly DNA cleavage.^66^ The CRISPR/Cas system functions through three main stages to recognize and destroy foreign DNA: adaptation, expression, and interference stage. CRISPR/Cas systems are natural immune mechanisms utilized by bacteria and archaea to combat viral invasions, subsequently harnessed by scientists as potent gene editing tools.^67,68^ This technology has widespread applications in biomedical research, agriculture, and other fields, significantly advancing the study and application of genetics.^69^

## 4. The application of CRISPR/Cas in molecular detection

CRISPR/Cas systems have witnessed a surge in their utilization in the field of diagnostics. As molecular diagnostic tools, they possess high sensitivity and specificity, capable of rapid testing, thereby reducing the dependence on expensive equipment and specialized personnel compared to traditional PCR techniques.^70^ CRISPR/Cas systems distinguish in detecting DNA or RNA molecules, particularly in rapid identification of pathogens by recognizing their specific DNA or RNA sequences.^71^ CRISPR/Cas systems are primarily applied in molecular detection due to their efficient nucleic acid recognition and detection capabilities. CRISPR/Cas systems demonstrate tremendous potential in detecting pathogen DNA or RNA due to their high specificity and sensitivity.^71^ Initially, CRISPR-Cas systems were developed for nucleic acid recognition in molecular diagnostics.^72^ Systems such as SHERLOCK and DETECTR represent significant applications of CRISPR/Cas technology in rapid diagnostic tools. Additionally, CRISPR/Cas systems can detect point mutations or single nucleotide variations, crucial for pathogen analysis during infection.^71^ Researchers have also employed signal amplification techniques to enhance the signal strength of CRISPR/Cas systems.^71^ These systems extend beyond nucleic acid detection into clinical and other application areas, including the use of Cas14 for diagnostics.^73^ Another important application of CRISPR/Cas systems is in constructing biosensing platforms for specific infectious disease detection and diagnosis.^74^ The continuous development of CRISPR/Cas technology has greatly advanced the development of molecular diagnostic systems.^70^

In summary, the utilization of CRISPR/Cas systems in molecular detection holds vast promise and is widely applicable.^75^ CRISPR/Cas systems possess characteristics such as high efficiency, specificity, and considerable scalability. Their integration into molecular diagnostics enhances detection sensitivity and specificity streamlines operational procedures, and lowers costs, thereby offering robust technical support for rapid and convenient pathogen detection. However, challenges persist in their clinical application, including the need for suitable delivery tools and concerns regarding toxicity, immunogenicity, and off-target effects.^76^ The application of CRISPR/Cas systems in molecular detection has transitioned from laboratory research to practical applications, demonstrating broad prospects and significant development potential. Nonetheless, overcoming challenges such as enhancing detection stability and reducing costs is crucial for broader adoption in practical clinical diagnostics.^77–79^

### 4.1 The structure and mechanism of CRISPR/Cas 9

The CRISPR/Cas9 system, originating from prokaryotic immune mechanisms, is a potent gene-editing tool defending against bacteriophages and conjugative plasmids.^80^ The core of the CRISPR/Cas9 system is the Cas9 protein, a single effector protein that introduces specific double-strand breaks at target DNA sequences guided by gRNA.^81^ CRISPR/Cas9 technology has revolutionized genome editing across species. Cas9 nuclease cleaves target DNA with the help of *trans*-activating CRISPR RNA (tracrRNA) and CRISPR RNA (crRNA), ensuring sequence specificity through Watson–Crick base pairing^26^ and recognizing adjacent PAM sequences for activation and cleavage.^82^ Cas9 protein includes essential regions for gRNA and target DNA binding.^83^ The simplicity and efficiency of CRISPR/Cas9 have fueled its broad use in genome engineering, influencing both DNA editing and gene regulation across species, significantly advancing biological research.

**Scheme 2:**
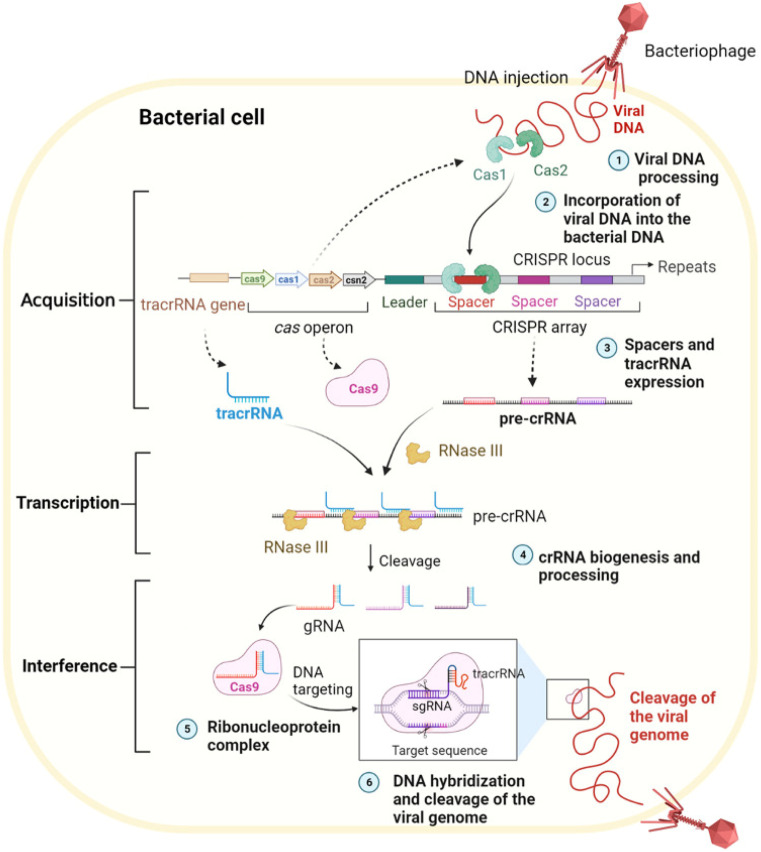
Biological mechanisms of the CRISPR/Cas9 system. This scheme has been reproduced from ref. 85 with permission from Du, *et al.* Copyright 2023.

**Scheme 3:**
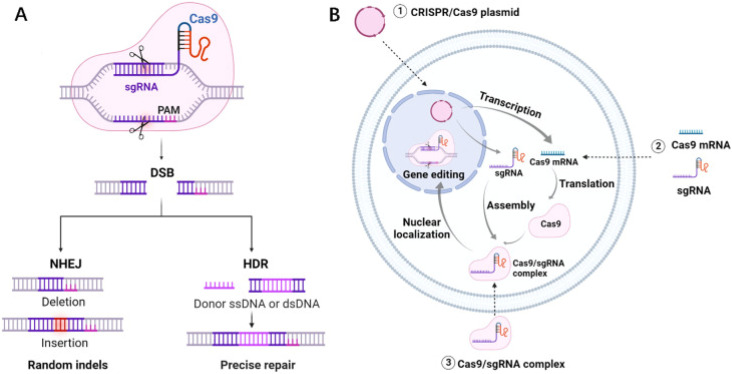
(A) Schematic diagram of the molecular mechanism by which the CRISPR/Cas9 system mediates gene editing. (B) Three forms of the CRISPR/Cas9 system. This scheme has been reproduced from ref. 85 with permission from Du, *et al.* Copyright 2023.

The CRISPR/Cas9 mechanism comprises acquisition, transcription, and interference stages: acquiring spacers from invaders, transcribing the CRISPR locus, and targeting viruses^84,85^ (as shown in the Schemes 2 and 3).

#### 4.1.1 The application of CRISPR/Cas9 system combined with DNA nanotechnology

CRISPR/Cas9 has become a novel tool for the precise detection of nucleic acids and other substances due to its efficiency and speed. Cas9, a programmable nucleic acid “nicking” tool, cleaves target ssDNA with high efficiency and simplicity. Guided by sgRNA, the Cas9 protein locates and cleaves the DNA strand near the protospacer adjacent motif (PAM) site. Recent diagnostic techniques based on CRISPR have gained attention for their sensitivity (from Cas's collateral cleavage), selectivity (*via* enzyme recognition), and versatility (through programmability).^86^ Combining CRISPR-Cas system with DNA nanotechnology can be used to detect nucleic acids, proteins, and small molecules. Utilizing these biosensors, it is possible to develop new point-of-care diagnostic methods for pathogen detection, genotyping, cancer mutation detection, and disease diagnosis.^26^

#### 4.1.2 The application of CRISPR/Cas9 system combined with two-dimensional DNA nanostructures

**Fig. 1:**
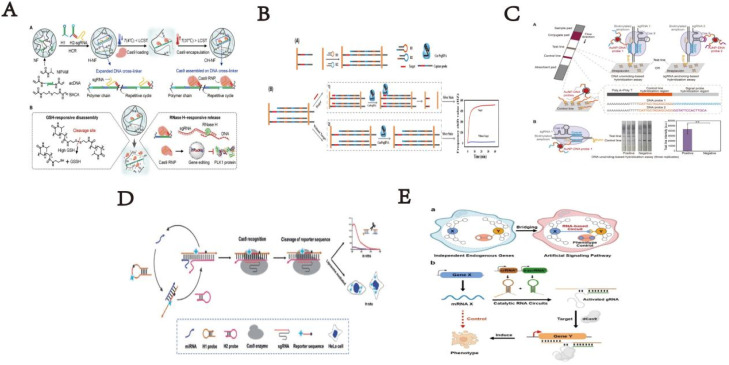
(A) Scheme of cascade dynamic assembly/disassembly of NF as a nanocarrier for the controlled delivery of Cas9 RNP. This figure has been reproduced from ref. 15 with permission from Song, *et al.* Copyright 2023. (B) Response mechanism of development of an MSPQC nucleic acid sensor based on CRISPR/Cas9 for the detection of *Mycobacterium tuberculosis*. This figure has been reproduced from ref. 16 with permission from Huang, *et al.* Copyright 2022. (C) Schematic illustrating the developed CASLFA method. This figure has been reproduced from ref. 17 with permission from Wang, *et al.* Copyright 2020. (D) Scheme of a sensitive and specific method for microRNA detection and *in situ* imaging based on a CRISPR-Cas9 modified CHA. This figure has been reproduced from ref. 18 with permission from Liu, *et al.* Copyright 2020. (E) Strategy of constructing RNA-based artificial signaling pathways between endogenous genes in mammalian cells. This figure has been reproduced from ref. 19 with permission from Wu, *et al.* Copyright 2024.

2D DNA structures *via* DNA nanotechnology form under specific conditions with stable, flexible DNA strands interactions. These 2D products formed by hybridization chain reaction (HCR), rolling ring amplification reaction (RCA), catalytic hairpin assembly (CHA), and exponential isothermal amplified strand displacement reaction (EXPAR). Combining CRISPR/Cas9 system with DNA nanotechnology in biosensors allows integrates small molecules (DNA, RNA, and proteins), boosting detection sensitivity and specificity. This greatly leverages the gene-editing capabilities, biological detection capabilities, and bio-delivery capabilities of the CRISPR/Cas9 system. For instance, Song *et al.* described dynamic assembly/disassembly of DNA nanoframeworks for controlled Cas9 ribonucleoprotein (RNP) delivery.^15^ Through HCR, sgRNA binds with the NF to form DNA-RNA complexes; the internal space of the NF expands, facilitating the loading of the Cas9 protein. In cancer cells, the overexpressed ribonuclease H (RNase H) digest RNA in DNA-RNA complexes, releasing Cas9 RNP for gene editing. Huang *et al.* proposed a novel MSPQC M.TB sensor based on the CRISPR/Cas9 system.^16^ This sensor can distinguish single base mismatches in the 10-base protospacer adjacent motif (PAM) region. In the proposed sensor, single-stranded DNA on gold interdigitated electrodes is used as a capture probe for the target and as an initiator for the HCR. CRISPR/Cas9 acts as the recognition element to identify the capture/target dsDNA. When the target is present, the capture probe hybridizes with the target to form dsDNA, which the CRISPR/Cas9 can recognize and cleave. The sensor targets specific sequence fragments of M.TB 16S rRNA for M.TB detection. The shortest detection time is 2.3 hours, with a detection limit (LOD) of 30 CFU mL^−1^, utilizing the CRISPR/Cas9 tool. Xusheng Wang and *et al.* have also developed a rapid, accurate, and portable nucleic acid detection platform that integrates CRISPR/Cas9 recognition technology into a lateral flow detection platform, minimizing the reliance on equipment and specialized personnel. Therefore, they named this technology CRISPR/Cas9-mediated lateral flow nucleic acid assay (CASLFA). CASLFA achieved a detection limit of a few hundred gene copies, comparable to PCR. Importantly, by adopting a sgRNA-anchored hybridization assay, the AuNP-DNA probe is universal, and the lateral flow device pre-assembled with the AuNP-DNA probe can be used to detect any target gene. The CASLFA method maintains high sensitivity while also exhibiting high specificity.^17^ CHA, an enzyme-free self-assembly signal amplification strategy, is noted for identifying DNA, RNA, and small molecules *in vitro* and *in vivo*. Many methods combining CRISPR-Cas9 system's specific cleavage activity with *in vitro* amplification strategies for detecting dsDNA and ssDNA. Liu *et al.* reported CRISPR-Cas9 and CHA for exosome miRNA and live cell imaging detection.^18^ The signal output modes of CHA are diverse, including commonly used electrochemical, fluorescent, and colorimetric methods, which exhibit high signal-to-noise ratios and strong universality. However, the specificity of CHA methods still requires further improvement. And Wu *et al.* engineered gene networks *via* synthetic signal pathways (ASP) to reprogram cell responses and phenotypes under different conditions, aiming for various diagnostic and therapeutic purposes. The research team utilized the CHA system combined with controllable CRISPR-Cas9 to convert signals triggering mRNA expression into target genes regulation. By introducing these RNA-based gene circuits, mammalian cells were endowed with autonomy, enabling response to ligand stimuli and RNA expression changes across cell types, controlling cell fate *via* ASPs linked to cellular apoptosis (Fig. 1).^19^

RCA has emerged as a highly specific isothermal gene amplification method. RCA can be conducted at a constant temperature in the presence of polymerase (30 °C or even room temperature) without the need for complex instruments. The high specificity of RCA reaction is mainly attributed to stringent parameter requirements, particularly the need for complete primer complementarity with the circular DNA template during initiation, rendering it ideal for single nucleotide polymorphism analysis. Liu *et al.* in their laboratory integrated RCA, CRISPR/Cas9, and CHA technologies to develop a miRNA detection method. In this method, the target miRNA circularizes to trigger RCA, yielding long single-stranded DNA products with repeated hairpin structures. Upon addition of complementary sequences, dsDNA forms. Cas9 enzyme facilitates cleavage of RCA products into hairpin probes, which, when unfolded by target miRNA, initiate CHA for signal generation.^20^ Extracellular vesicle (EV)-derived microRNAs (miRNAs) play a crucial role in disease diagnosis and prognosis assessment due to their multiplex detection capabilities. Here, Wang *et al.* developed a highly specific nucleic acid detection platform.^21^ Integrating CRISPR/Cas system and RCA technology, this platform quantitatively detects multiple EV-derived miRNAs isothermally. Specifically, the method achieves single-base resolution *via* dual-specificity recognition using hairpin probe-mediated ligation and PAM-triggered cleavage. The robustness of the method was validated by high consistency in detecting EV-derived miRNA abundance using RCA-assisted CRISPR/Cas9 cleavage (RACE) and RT-qPCR in cancer cells and lung cancer patients. This highlights its potential in screening, diagnosing, and prognosticating various diseases. In summary, RACE is a powerful tool for multiplex, specific nucleic acid detection in point-of-care diagnostics and on-site analysis. Giving increasing concerns about food safety, rapid identification of *Staphylococcus aureus* (*S. aureus*) is of significant importance. Here, Zhen *et al.* have designed a novel electrochemical biosensor^22^ based on the CRISPR/Cas9 system and RCA-assisted “silver chain” linking on gold interdigitated electrodes (Au-IDE). This sensor utilizes RCA to generate long DNA chains spanning the Au-IDE, with CRISPR/Cas9 serve as the recognition element to identify capture/target dsDNA. Additionally, silver staining technology was employed to enhance detection sensitivity. The sensor detects *Staphylococcus aureus* by monitoring impedance changes caused by the connection or disruption of silver chains on the Au-IDE, achieving a detection limit (LOD) of 7 CFU mL^−1^ within 1.5 hours. Importantly, this sensor successfully identifies *Staphylococcus aureus* in real food samples, highlighting its potential for food monitoring applications.

**Fig. 2:**
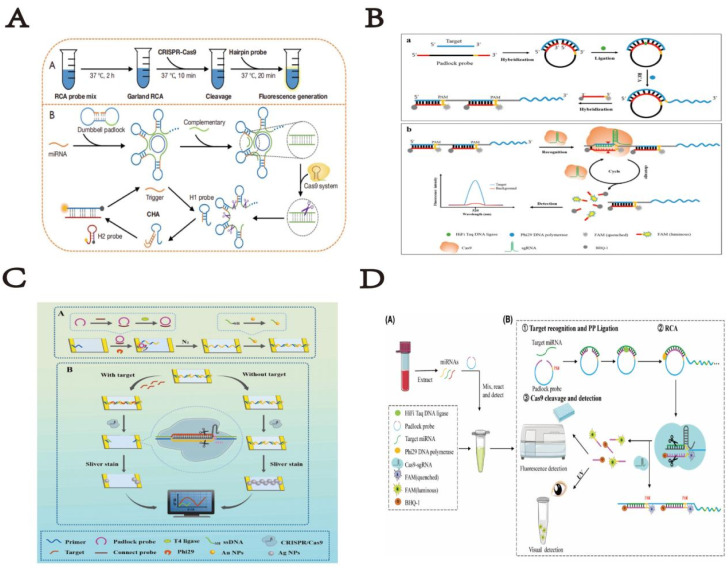
(A) Scheme of CRISPR/Cas9-assisted garland RCA-based miRNA detection approach. This figure has been reproduced from ref. 20 with permission from Liu, *et al.* Copyright 2022. (B) Schematic reaction mechanism of RACE. This figure has been reproduced from ref. 21 with permission from Wang, *et al.* Copyright 2020. (C) Schematic illustration of a supersensitive electrochemical sensor based on RCA amplification-assisted “silver chain”-linked gold interdigital electrodes and CRISPR/Cas9 for the detection of *Staphylococcus aureus* in food. This figure has been reproduced from ref. 22 with permission from Zhen, *et al.* Copyright 2024. (D) Scheme of parasite-derived microRNA let-7-5p detection for metacestodiasis based on rolling circular amplification assisted CRISPR/Cas9. This figure has been reproduced from ref. 23 with permission from Wang, *et al.* Copyright 2024.

In other ecological detection studies, Wang *et al.* utilized RCA assisted CRISPR/Cas9 detection method to investigate miRNA let-7-5p derived from parasites and other sources.^23^ The team used a series of diluted let-7 standard samples to compare the limits of detection (LOD) among qPCR, RCA, and RCA-assisted CRISPR/Cas9 methods. The results indicated that RCA-assisted CRISPR/Cas9 assay could effectively differentiate let-7 from tapeworms and other species, achieving an LOD of 10 aM. These findings highlight that the RCA-assisted CRISPR/Cas9 method for let-7-5p detection is sensitive and specific, serving as a universal diagnostic tool for diseases like tapeworm infections, particularly suitable for early diagnosis (15 dpi). In this study, compared to traditional qPCR and RCA methods, the RCA-assisted CRISPR/Cas9 method for analyzing let-7 exhibited superior sensitivity. Notably, sensitivity is paramount in detecting samples with low abundances, notably miRNAs, particularly during the early stages of pathogen infection diagnosis. Despite its advantages, this new method still has limitations. The experimental process involves three steps, posing a potential risk of cross-contamination. Therefore, future research should prioritize developing single-tube detection methods to reduce the processing time and enhancing accuracy. Additionally, this method possesses the potential for detecting various other arthropod species. Given the relatively low incidence of human arthropod infections, this method holds promise for early diagnosis of human cysticercosis and trichinellosis. The integration of this methodology with radiological techniques holds the potential to significantly augment its diagnostic accuracy (Fig. 2).

EXPAR is a robust nucleic acid amplification technique involving polymerase-directed chain extension and single-strand cleavage induced by nicking endonucleases (NEase), which has proven effective in nucleic acid and protein analysis. Compared to other isothermal amplification methods, EXPAR offers superior amplification efficiency and rapid kinetics. Here, Huang *et al.* have developed a CRISPR/Cas9-triggered exponential amplification method (CAS-EXPAR),^24^ which combines the site-specific cleavage of Cas9/sgRNA with the rapid amplification kinetics of EXPAR. This method eliminates the need for exogenous primers that may lead to non-specific triggering reactions. Instead, “primers” are generated through site-specific cleavage of the target DNA sequence and accumulate during the reaction. Using real-time fluorescence intensity analysis, CAS-EXPAR achieves sensitive DNA detection within 1 hour. In addition, by combining bisulfite conversion to convert single-base methylation into single-base mutations, this strategy can also achieve site-specific DNA methylation detection. CAS-EXPAR utilizes target DNA fragments generated by CRISPR/Cas9 cleavage as primers for the exponential amplification reaction, which produces large amounts of DNA copies monitored in real-time by fluorescence detection. This strategy combines the advantages of CRISPR/Cas9 and exponential amplification, featuring high specificity and rapid amplification kinetics. The detection limit of this strategy is 0.82 amol, demonstrating excellent specificity in distinguishing single-base mismatches. Song *et al.* described a new method for detecting DNA mutations using EXPAR triggered by clustered regularly interspaced short palindromic repeats (CRISPR), termed CRISPR-EXPAR.^25^ The research team designed a CRISPR system composed of two Cas9/sgRNA complexes to cleave and identify a specific mutation region within the target DNA. This facilitates EXPAR through iterative extension and cleavage reactions with minimal repeats. Based on this design principle, they successfully identified mutations in the human epidermal growth factor receptor 2 (HER2) gene, achieving a detection limit as low as 437 aM, with excellent specificity. This technology can serve as a novel isothermal method by redesigning single-guide RNA (sgRNA) to effectively recognize various mutation target sites.

Circulating tumor DNA (ctDNA) plays a crucial role in the early diagnosis and prognosis of various cancers, serving as a reliable biomarker for predicting treatment responses. However, the low abundance of ctDNA in peripheral blood and the high background of wild-type DNA affect the precise and specific measurement of ctDNA. In this significant study, Mei Chen *et al.* developed a novel three-dimensional GR/AuPtPd nano-flower sensing platform based on Entropy-Driven Strand Displacement Reaction (ESDR) triggered by CRISPR/Cas9 cleavage, for effective detection of ctDNA.^26^ Due to the complex operational steps and stringent reaction conditions required for ESDR amplification, it was used to detect mutations in epidermal growth factor receptor (EGFR) ctDNA. This method combines the site-specific cleavage advantage of ‘gene scissors’ Cas9/sgRNA with the rapid amplification kinetics of ESDR, achieving not only high amplification efficiency but also high specificity in distinguishing single nucleotide mismatches. The diagram below details the guiding principles of this technology. Based on the proven effectiveness of the three-dimensional GR/AuPtPd hybrid nano-flower as an excellent electrochemical sensing platform, the electrochemical biosensor based on three-dimensional GR/AuPtPd nano-flower demonstrates high specificity and excellent performance in human serum detection. It significantly enhances the sensitivity of detecting ctDNA using CRISPR/Cas9-triggered ESDR. This innovative strategy serves as a multifunctional DNA detection method for biological analysis and clinical diagnostics, providing a new paradigm for efficient ctDNA detection and demonstrating enormous potential in clinical and diagnostic applications.

**Fig. 3:**
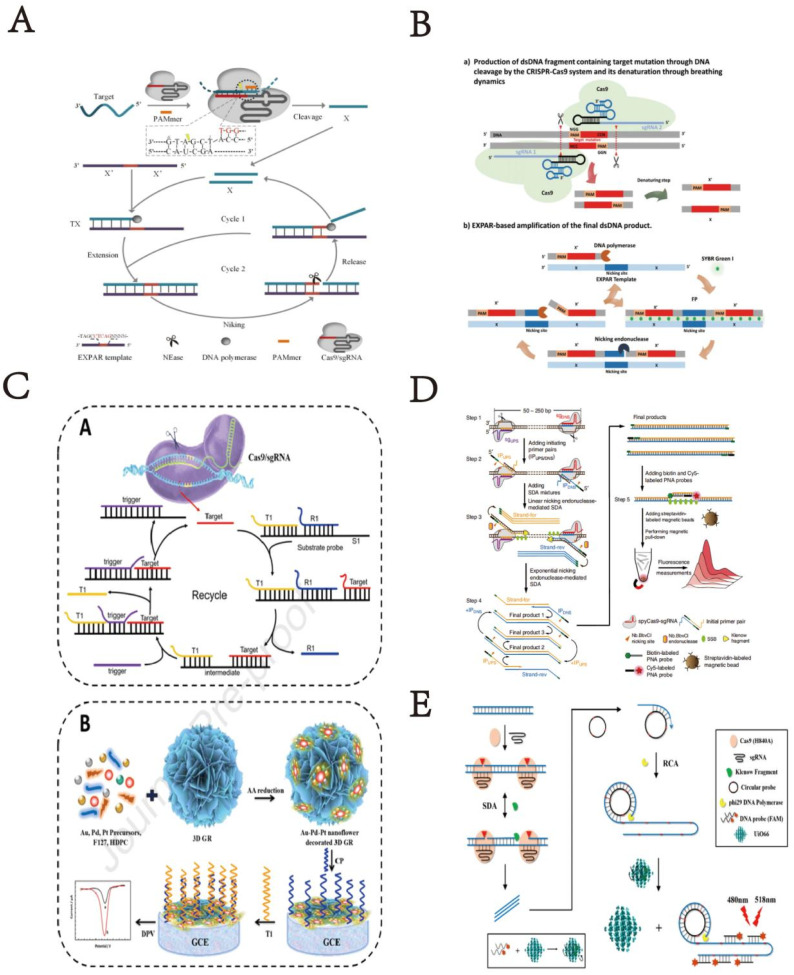
(A) Schematic illustration of CRISPR/Cas9 triggered isothermal amplification for site-specific nucleic acid detection. This figure has been reproduced from ref. 24 with permission from Huang, *et al.* Copyright 2018. (B) Schematic illustration of the CRISPR-EXPAR strategy for the detection of target mutation. This figure has been reproduced from ref. 25 with permission from Song, *et al.* Copyright 2021. (C) Schematic of the principle of the CRISPR/Cas9-triggered ESDR based on a 118 3D GR/AuPtPd nanoflower biosensor. This figure has been reproduced from ref. 26 with permission from Chen, *et al.* Copyright 2021. (D) Schematic reaction mechanism of CRISDA for ultrasensitive DNA Detection. This figure has been reproduced from ref. 27 with permission from Zhou, *et al.* Copyright 2018. (E) Scheme of CRISPR-Cas9 triggered SDA-RCA method based on UiO66 platform. This figure has been reproduced from ref. 28 with permission from Sun, *et al.* Copyright 2020.

DNA isothermal amplification technologies have more great advantages than traditional PCR techniques in diagnostic applications. Such as Zhou *et al.* reported a CRISPR-Cas9-triggered nucleic acid endonuclease – mediated strand displacement amplification (SDA) method (abbreviated as CRISDA),^27^ used for sensitive amplification and detection of double-stranded DNA (dsDNA). This technology fully utilizes the high sensitivity, strict specificity, and unique conformational rearrangement of CRISPR effectors in recognizing target DNA. The method is combined with robust peptide nucleic acid (PNA) invasion-mediated endpoint measurements, achieving sub-atto-molar sensitivity and single-base specificity even in complex sample backgrounds. After a series of concept validation studies, they demonstrated the ultra-high sensitivity detection capability of CRISDA by specifically amplifying and detecting target DNA segments in both human and transgenic soybean MON87705 genomes. Finally, by integrating CRISDA technology with Cas9-mediated target enrichment methods, they showcased the versatility of CRISDA, highlighting its sub-atto-molar sensitivity. CRISDA thus emerges as a powerful tool for nucleic acid ultra-sensitive detection and diagnostics. In recent decades, methods used to detect *Escherichia coli* O157:H7 have included traditional culture methods, real-time PCR, ELISA (enzyme-linked immunosorbent assay), and others. However, these traditional detection methods are time-consuming, complex, and low sensitivity. Therefore, researchers have developed various methods, including fluorescence, chemiluminescence, and electrochemical detection combined with various biosensors, to detect *Escherichia coli* O157:H7. Among all these methods, fluorescence is widely used due to its rapid analysis and high sensitivity. Therefore, Sun *et al.* developed a two-step isothermal amplification method triggered by CRISPR-Cas9 for fluorescence detection of *Escherichia coli* O157:H7.^28^ It is reported that *E. coli* O157:H7 is an important pathogen causing severe infections and economic losses. In the study, the CRISPR-Cas9 system recognized and cleaved the target virulence gene sequence, triggering strand displacement amplification and RCA. After amplification, the abundant products hybridized with probes, and the fluorescence of the probes was quenched on a metal–organic framework platform, thereby restoring fluorescence at atypical excitation/emission wavelengths of 480/518 nm. This method exhibits high sensitivity with a detection limit of 4.0 × 10^1^ CFU mL^−1^ and a detection range from 1.3 × 10^2^ CFU mL^−1^ to 6.5 × 10^4^ CFU mL^−1^. Moreover, the detection method demonstrates significant specificity and is applicable to real samples, ensuring high accuracy, holding great potential for applications in bacterial detection, food safety monitoring, or clinical diagnostics (Fig. 3).

#### 4.1.3 The application of CRISPR/Cas9 system combined with three-dimensional DNA nanostructures

With the rapid development of DNA nanotechnology, DNA nanostructures have shown enormous potential applications in fields such as biosensing, bioimaging, drug delivery, cell biology, and materials manufacturing.^87^ Typical three-dimensional DNA nanostructures include G-quadruplexes, DNA tetrahedrons, DNA dendrimers, amorphous structures like DNA hydrogels, and DNA origami structures. DNA, as an ideal sensing molecule, can be precisely designed into nanostructures due to its programmability, assembling into spatial structures with specific sizes and shapes. These structures are not only specific but also robust and durable, capable of functioning under a wide range of biologically relevant temperatures and conditions.^88–90^ In recent years, the presence of G4 structures in the human genome has been directly visualized using antibodies against G4 structures and fluorescent G4 probes.^91^ DNA enzymes, based on DNA, are important catalysts known for their excellent activity, programmability, signal amplification through catalytic turnover, high chemical stability, simplicity of synthesis, and ease of modification, making them widely applicable.^92^ Given these significant characteristics, hemin/G-quadruplex DNA enzymes have been widely used in electrochemical, colorimetric, chemiluminescent sensors, and biosensors for detecting various targets. In this context, we focus on the synergistic application of the CRISPR system with G-quadruplexes. The design of G-quadruplex and similar structures enhances the gene editing capabilities of the CRISPR system, yet their application together in biosensors for target detection is less explored.

CRISPR technology holds great promise in gene regulation and editing. Deng *et al.* designed a light switch on G-quadruplex gRNA (GqRNA), embedding bis-azobenzene derivatives (AZD^++^) to precisely control gene editing and expression. Their research indicates that rational design of G-quadruplex on crRNA provides higher stability and sequence recognition specificity compared to unmodified sgRNA. Therefore, the proposed light-reversible mode strategy offers a new opportunity for regulating CRISPR-Cas9, enhancing its safety and applicability.^29^ Although Cas9 can effectively bind and cleave DNA under *in vitro* conditions, its activity varies significantly in cells due to differences in target sites. This variability depends on genomic locus disparities and the availability of sufficient Cas9/single guide RNA (sgRNA) complexes for cleavage. To date, most methods rely on Cas9 protein engineering or base modifications in sgRNA sequences to enhance CRISPR/Cas9 activity. Smita Nahar *et al.* demonstrated that structure-based rational design of sgRNA can improve Cas9 cleavage efficiency *in vivo*. By appending a naturally occurring RNA G-quadruplex motif at the 5′ end of sgRNA, stability and target cleavage efficiency were enhanced in zebrafish embryos without inducing off-target effects. This highlights its value in designing better and more optimized genome editing triggers.^30^ Accurate and effective detection of single-stranded nucleic acids is crucial in disease diagnostics and pathological research. Therefore, Wang *et al.* developed a PAM-assisted CRISPR/Cas9 system-mediated G4-EXPAR (Cas-G4EX) strategy,^31^ which integrates sequence-dependent cleavage of the CRISPR/Cas9 system with the high efficiency of exponential amplification. Cas-G4EX integrates the advantages of CRISPR/Cas9 and EXPAR, exhibiting excellent site-specific recognition capability and high-performance amplification efficiency. EXPAR products are short ssDNA chains rich in G, capable of forming G-quadruplex structures in the presence of hemin. G4/hemin mimics peroxidase activity, catalyzing the ABTS-H_2_O_2_ system, thereby displaying bright green color. Meanwhile, the programmability of the CRISPR/Cas9 system makes this method a universal detection paradigm for any ssRNA or ssDNA. Cas-G4EX also demonstrates good discriminatory ability for single-base mutations in single-stranded nucleic acids. Therefore, the Cas-G4EX detection method provides a promising platform for molecular diagnostics and pathological analysis applications.

Song *et al.* reported a multifunctional DNA/upconversion nanoparticle (UCNP) complex based on RCA, which synergistically delivers Cas9 RNP, hemin, and PP for combined photodynamic therapy (PDT). High gene editing efficiency and efficient PDT were achieved both *in vitro* and *in vivo*, indicating the clinical trial potential of UCND. The multifunctional and intelligent DNA/UCNP complex overcomes limitations and barriers associated with PDT, thus poised to meet the demands of precision medicine.^32^

**Fig. 4:**
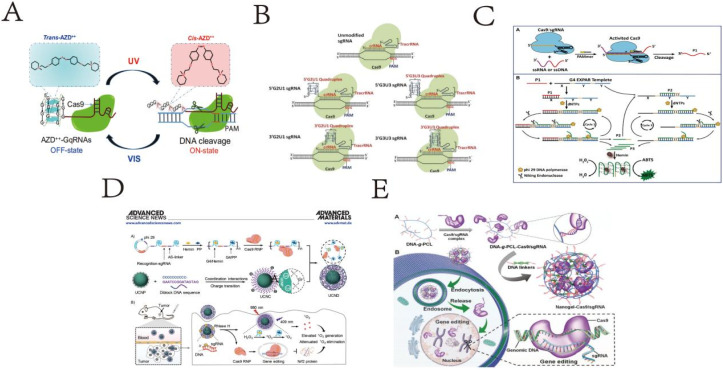
(A) Schematic illustration of G-quadruplex-based CRISPR photoswitch for spatiotemporal control of genomic modulation. This figure has been reproduced from ref. 29 with permission from Deng, *et al.* Copyright 2023. (B) Representation of various Cas9–sgRNA complexes. This figure has been reproduced from ref. 30 with permission from Nahar, *et al.* Copyright 2018. (C) Schematic illustration of Cas-G4EX assay for colorimetric detection of ssRNA and ssDNA. This figure has been reproduced from ref. 31 with permission from Wang, *et al.* Copyright 2021. (D) Scheme of an RCA-based multifunctional DNA/UCNP complex for the controlled co-delivery of Cas9 RNP, hemin, and PP for synergistic photodynamic therapy. This figure has been reproduced from ref. 32 with permission from Song, *et al.* Copyright 2024. (E) Schematic illustration of the Cas9/sgRNA-embedded nucleic acid nanogel formation and its delivery *in vitro*. This figure has been reproduced from ref. 33 with permission from Ding, *et al.* Copyright 2019.

Using DNA as the sole component, skeleton, or crosslinking agent of hydrogels, pure DNA hydrogels are formed through chemical interactions (where chemical bonds act as crosslinking points) or physical interactions (non-covalent interactions such as hydrogen bonds, van der Waals forces, or interlacing of DNA molecular chains). Hydrogels have advantages such as large surface area and high biocompatibility, making them efficient for sensing and other applications. Ding *et al.* developed a CRISPR/Cas9 delivery platform based on non-cationic nanogels.^33^ In this platform, DNA-g-PCL brushes loaded with Cas9/sgRNA are crosslinked through nucleic acid hybridization to form nanogels. After embedding in the nanogels, the physiological stability of Cas9/sgRNA is significantly enhanced due to the protective compact structure of the nanogels. This nanogel-based CRISPR/Cas9 delivery system is the first example using non-cationic DNA nanostructures as carriers for gene editing tools. Embedding in nanogels significantly enhances the physiological stability of Cas9/sgRNA gene editing tools (Fig. 4).

### 4.2 The structure and mechanism of CRISPR/Cas12

The CRISPR/Cas12 system is an adaptive immune mechanism found in bacteria and archaea, used for editing and detection by targeting specific DNA sequences in humans and other organisms. Structurally, the CRISPR/Cas12 system exhibits several significant features. Unlike Cas9, Cas12a (also known as Cpf1) and Cas12b (also known as C2c1) proteins are typically smaller, about half the size of Cas12a.^93^ These compact Cas12 proteins display structural diversity, offering possibilities for expanding the CRISPR toolbox. Furthermore, the fundamental principle of CRISPR-Cas systems involves the assembly of CRISPR-derived transcripts (crRNA) with Cas proteins to target and cleave complementary nucleic acids.^94^ The various subtypes of Cas12, including Cas12a, Cas12b, Cas12c, and Cas12d, further broaden its application prospects.^95^ Cas12 proteins demonstrate high specificity and efficiency in recognizing and cleaving double-stranded DNA through their unique cleavage mechanism and protein structure, allowing for targeted binding to specific sites and local unwinding of the DNA helix^94^^.^ Cas12 proteins also exhibit non-specific *trans*-cleavage activity on single-stranded DNA, a feature that is particularly useful in molecular diagnostics.^96^

**Scheme 4:**
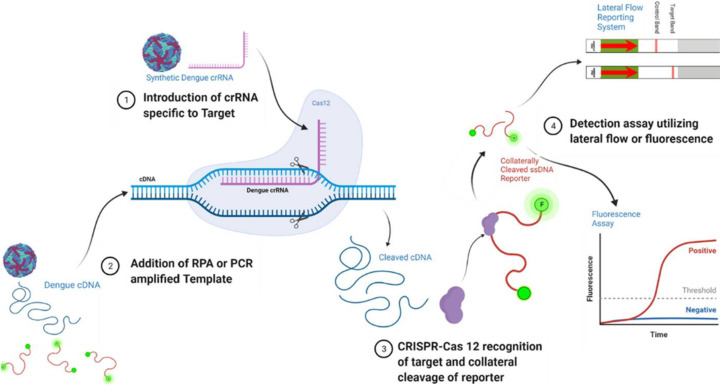
Overview of CRISPR-Cas 12 based assay approaches. This scheme has been reproduced from ref. 99 with permission from Mann, *et al.* Copyright 2022.

The diversity and unique structure of the CRISPR-Cas12 system make it a powerful tool for gene editing and nucleic acid detection. These systems not only efficiently perform gene editing but also maintain activity across a wide range of temperatures, demonstrating high specificity and minimal off-target effects in mammalian genome engineering.^97,98^ Structural studies of the CRISPR/Cas12 system reveal its enormous potential as a versatile tool for gene editing and molecular detection. Understanding the detailed structure and functional characteristics of the CRISPR/Cas12 system enables scientists to further optimize and develop new CRISPR-Cas tools to meet the diverse needs of biomedical and biotechnological applications. The biological mechanisms of the CRISPR/Cas12 system can be divided into several stages^99^ (as shown in the scheme below). In conclusion, the CRISPR/Cas12 system is a versatile tool for gene editing and molecular diagnostics, with its wide-ranging applications and unique mechanisms playing a significant role in biomedical research and clinical applications (Scheme 4).

#### 4.2.1 The application of CRISPR/Cas12 system combined with DNA nanotechnology

CRISPR/Cas systems are among the most powerful molecular scissors, capable of precisely and specifically identifying and cleaving target nucleic acids in a simple and flexible manner, a capability unmatched by other molecular scissors like restriction enzymes and zinc finger nucleases. When used for analysis and diagnostics, CRISPR/Cas systems leverage their unique characteristics to exhibit significant advantages. Firstly, CRISPR/Cas systems specifically recognize target nucleic acids through the 20-base pair spacer region of the crRNA, ensuring sequence-specific detection and site-specific imaging, while minimizing off-target binding or cleavage. Moreover, crRNA can be chemically synthesized in large quantities and modified, greatly accelerating the screening and application of crRNAs with adjustable specificity (sensitive or tolerant to single nucleotide mutations). Secondly, the CRISPR/Cas system can recognize dsDNA without denaturation. This unique binding property allows the CRISPR/Cas system to directly capture and detect target genes on various optical and electrochemical detection platforms, as well as enabling *in situ* imaging of genomic loci in live cells. Thirdly, the ease of recognition of dsDNA amplicons facilitates the integration of the CRISPR/Cas system with other nucleic acid amplification method, including PCR, LAMP, and RPA, developing ultra-sensitive nucleic acid detection methods. Furthermore, the CRISPR/Cas system boasts remarkable flexibility and modularity, featuring diverse nuclease activity modes. By selecting the appropriate Cas mechanism, researchers can detect a broad spectrum of nucleic acid targets, encompassing dsDNA, ssDNA, and RNA, as evidenced by previous studies.^100,101^

In recent years, the distinctive *trans*-cleavage activity of Cas12a has been harnessed extensively in the development of Cas12a detection techniques with different signal output methods to achieve simple, ultra-sensitive, and portable detection of targets.

#### 4.2.2 The application of CRISPR/Cas12 system combined with two-dimensional DNA nanostructures

CRISPR/Cas12 systems combined with DNA nanotechnology flexibly recognize nucleic acids, proteins, metal ions, and other substances in biosensors, greatly enhancing detection sensitivity, specificity, and other performance metrics. Simultaneously, they significantly leverage the gene editing capabilities and biosensing detection capabilities of CRISPR/Cas12 systems. The following section mainly elaborates on the technical applications of CRISPR/Cas12 systems combined with 2D DNA nanostructures such as HCR, CHA, RCA and so on.

Cheng *et al.* proposed a lateral flow assay technology based on chain hybridization strategy in their research,^34^ named CHLFA, a nucleic acid hybridization-based lateral flow assay platform. They integrated CRISPR technology with CHLFA to construct the CRISPR-CHLFA visual gene detection system. Based on this research, the developed CRISPR system and CHLFA platform demonstrated excellent performance in visual detection of SARS-CoV-2 and MTB clinical specimens. This work primarily showcases the CRISPR-CHLFA concept based on the Cas12a system, and the feasibility of CRISPR-CHLFA platform based on Cas13a has also been demonstrated, indicating that CRISPR-CHLFA can serve as a universal, multifunctional biosensing platform for point-of-care testing (POCT). Additionally, Zhang *et al.* developed a visual detection method based on AuNP, which combines Cas12a reverse transcription-assisted loop-mediated isothermal amplification (RT-LAMP) called CLAP detection method.^35^ CLAP analysis is easy to operate and scalable for high-throughput detection. This is the first colorimetric assay based on AuNP, combined with Cas12 and RT-LAMP, suitable for on-site diagnosis of COVID-19.

Polychlorinated biphenyls (PCBs) are ubiquitous persistent organic pollutants that have harmful effects on environmental safety and human health. In response, Deng *et al.* proposed a logic gate biosensing platform for simultaneous detection of multiple PCBs.^36^ The team designed a CHA module to convert and amplify each trigger DNA into programmable DNA duplexes, thereby initiating the reverse cleavage activity of CRISPR/Cas12a to achieve signal output. This biosensing platform offers advantages such as multiple input combinations, intuitive digital output, high flexibility, and scalability, making it highly promising for intelligent detection of various PCBs.

Current tools for detecting genetically modified crops, such as polymerase chain reaction (PCR), require specialized equipment and complex operations. Xiaoying Zhu and colleagues introduced a clustered regularly interspaced short palindromic repeats (CRISPR)/Cas system,^37^ utilizing isothermal amplification as the amplification reporter gene for analyzing transgenes. This approach allows for isothermal and label-free detection of transgenic crops. To enhance detection sensitivity, researchers employed RCA, designing RCA primers as substrates for Cas12a cleavage to monitor transgene recognition. This assay, named isoCRISPR, enables label-free and isothermal amplification detection of transgenic maize. isoCRISPR detection enriches the toolbox for identifying genetically modified crops and expands the application of CRISPR/Cas in food authenticity and safety.

**Fig. 5:**
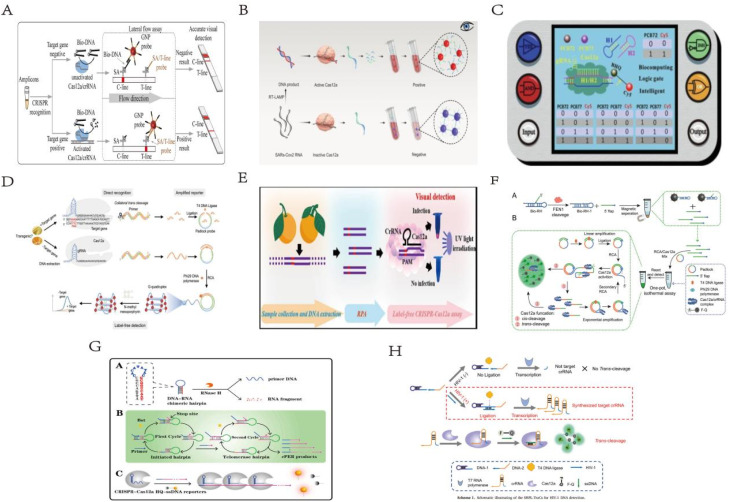
(A) Scheme of chain hybridization-based CRISPR-lateral flow assay enables accurate gene visual detection. This figure has been reproduced from ref. 34 with permission from Cheng, *et al.* Copyright 2021. (B) Schematic of the CLAP assay for detecting SARS-CoV-2 by the naked-eye readout. This figure has been reproduced from ref. 35 with permission from Zhang, *et al.* Copyright 2021. (C) Scheme of integrating CRISPR-Cas12a with CHA as a logic gate biosensing platform for the detection of polychlorinated biphenyls in water samples. This figure has been reproduced from ref. 36 with permission from Deng, *et al.* Copyright 2023. (D) Scheme of label-free detection of transgenic crops using an isothermal amplification reporting CRISPR/Cas12 assay. This figure has been reproduced from ref. 37 with permission from Zhu, *et al.* Copyright 2022. (E) Scheme of integrating CRISPR-Cas12a and rolling circle-amplified G-quadruplex for naked-eye fluorescent “off-on” detection of citrus *Alternaria*. This figure has been reproduced from ref. 38 with permission from Ma, *et al.* Copyright 2024. (F) Scheme of ultra-sensitive fluorescence biosensor with rolling circle amplification and CRISPR/Cas12a one-pot system for FEN1 detection. This figure has been reproduced from ref. 39 with permission from He, *et al.* Copyright 2025. (G) The working mechanism of the RNase H activity assay combining cPER with CRISPR/Cas12a. This figure has been reproduced from ref. 40 with permission from Xie, *et al.* Copyright 2022. (H) Schematic illustrating of the SRPL-TraCs for HIV-1 DNA detection. This figure has been reproduced from ref. 41 with permission from Song, *et al.* Copyright 2023.

Citrus *Alternaria*, a plant pathogen spread globally, easily triggers citrus brown spot disease and metabolizes mycotoxins. Lanrui Ma *et al.* introduced a ‘label-free’ and ‘on-site’ visual fluorescence detection method for citrus *Alternaria* brown spot disease based on CRISPR-Cas12a and RCA.^38^ This method uses ssDNA complementary to RCA primers as reverse cleavage substrates for CRISPR-Cas12a, cleverly combining CRISPR-Cas12a and RCA amplification of G-quadruplex structures. Using a portable light source for excitation, the experiment successfully distinguished *Alternaria* from other citrus pathogens, demonstrating the method's specificity. Validation of the method's practicality was achieved by analyzing cultured *Alternaria* and actual citrus leaf and fruit samples. Xiang-Lan He *et al.* have constructed a cascade signal amplification fluorescence biosensor for ultra-sensitive and rapid detection of FEN1. This biosensor relied on FEN1-induced production of the 5'flap DNA, and combined rolling circle amplification (RCA) and CRISPR/Cas12a one-pot system (RCOS). By utilizing branched dsDNA substrates to provoke FEN1 activity, the 5'flap DNA was cleaved and isolated through magnetic separation. By combining RCA and CRISPR/Cas12a one-pot cascade signal amplification, the detection signal was remarkable enhanced. The RCOS exhibited excellent sensitivity with a limit of detection (LOD) of 4.1 × 10^−7^ U μL^−1^, which was more sensitive and expeditious than many other approaches.^39^ In addition to the application of 2D DNA nanotechnology combined with CRISPR Cas12 system mentioned above, other 2D DNA nanotechnology also plays an important role in detecting biomolecular markers, like Zuowei Xie *et al.* introduce a new signal amplification strategy based on cascade primer exchange reaction (cPER) and CRISPR/Cas12a system for sensitive and specific analysis of RNase H activity. In the presence of RNase H, the RNA fragment of DR HP was specifically degraded and the blocked primer DNA was released. The process of enzymatic hydrolysis of substrate hairpin and cyclic signal amplification was completed in a one-step method under isothermal conditions, nriching many activator strands to initiate *trans*-cleavage of CRISPR/Cas system, Furthermore, inhibitor screening experiments and real samples analysis in human serum and cell extracts further verified that the proposed strategy possessed great potential in the application for biological enzyme research,^40^ and Yong-Li Song^41^ and other researches have developed a selective recognition proximity ligation and signal amplification with T7 transcription and CRISPR/Cas12a system, SRPL-TraCs, for highly sensitive and selective detecting HIV-1 DNA in homogeneous reaction. The T7 transcription accumulated a large amount of crRNA *in situ*, which were incorporated into the CRISPR/Cas12a system to construct cascade signal amplification strategies for target detection. Additionally, the detection system can achieve satisfactory reproducibility and reliability. Considering its high sensitivity, selectivity, and user-friendliness, maybe the SRPL-TraCs will be a promising tool for clinical diagnostics in the quantitative analysis of nucleic acids and fascinating biological molecule (Fig. 5).

#### 4.2.3 The application of the CRISPR/Cas12 system combined with three-dimensional DNA nanostructures

Among various signal transducers tested in nanopore experiments, DNA nanostructures with precise shapes and adjustable sizes have the potential to become the most widely applicable transducers. DNA nanostructures with certain hardness and uniform size can be assembled according to specific apertures. Their surfaces can be modified with different nucleic acid probes, small molecules, peptides, or protein antibodies to selectively bind to targets. The following section primarily elaborates on the technical applications of the CRISPR/Cas12 system combined with DNA tetrahedrons, G-quadruplexes, and other nanostructures.

CRISPR-Cas12a has proven to be an excellent signal transduction system. CRISPR-Cas12a is an RNA-guided enzyme that can specifically bind to and cleave target DNA. However, its application in nanosensing has only recently been reported. Zhang *et al.*^42^ expanded the application scope of CRISPR-Cas12a transduction methods through the innovative integration of the recently verified DNA tetrahedron as a signal transducer. The DNA tetrahedron was linked to magnetic beads *via* single-stranded DNA, while the CRISPR-Cas12a system serves to connect the DNA tetrahedron with the target DNA. Leveraging the inherent characteristics of the CRISPR-Cas12a system, this sensor ensures high sensitivity and specificity. Sensors based on the CRISPR-Cas12a transduction mechanism exhibit versatility in their adaptability to diverse, making them promising for practical diagnostic applications.

Early diagnosis of parasitic pathogens can significantly reduce medical, economic, and social burdens. Gao *et al.* reported the use of G4 DNAzyme as a reporter gene for CRISPR/Cas12, constructing a label-free detection method^43^ capable of early diagnosing infections of *Moniliformis moniliformis* within single cells. This detection method, known as GsubCas12, combines the *trans*-cleavage of Cas12a with catalytic oxidation using G4 DNAzyme, enabling detection of as few as 3.1 parasites. Moreover, compared to chemically labeled nucleic acid probes, the introduction of G4 DNAzyme proves more effective. Additionally, this assay does not require complex equipment for thermal cycling conditions. Benefiting from high programmability, GsubCas12 detection serves as a versatile platform for detecting pathogenic parasites, showing promise for diagnosing parasitic diseases in resource-limited areas.

Xia *et al.*, have developed an unlabeled high-sensitivity detection method for *Salmonella enterica* based on the G-quadruplex probe CRISPR-Cas12 system (referred to as G-CRISPR-Cas).^44^ The G-quadruplex probe serves as a substrate for Cas12a, enabling label-free analysis of foodborne pathogens. Due to the amplification process induced by loop-mediated isothermal amplification (LAMP), the G-CRISPR-Cas detection method can detect intestinal pathogens down to 20 CFU. G-CRISPR-Cas detection can be conducted in a single tube under isothermal conditions, making it suitable for on-site diagnosis of foodborne pathogen infections or contamination outside the laboratory. Therefore, the G-CRISPR-Cas analysis method holds promise for diagnosing foodborne pathogen infections or contamination in slaughterhouses, markets, and other outdoor settings. Ultimately, the G-CRISPR-Cas method was applied to study the colonization of intestinal streptococci in chicken intestines and organs.

**Fig. 6:**
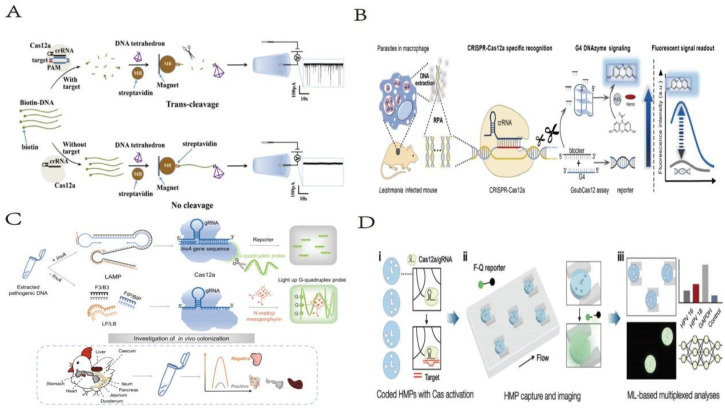
(A) Schematic illustration of a solid-state nanopore sensor using a DNA tetrahedron as a signal transducer based on the CRISPR-cas12a conversion mechanism. This figure has been reproduced from ref. 42 with permission from Zhang, *et al.* Copyright 2022. (B) Schematic diagram of GsubCas12 assay for detection of pathogenic parasites. This figure has been reproduced from ref. 43 with permission from Gao, *et al.* Copyright 2022. (C) Working principle of G-CRISPR-Cas assay and its application for label-free analysis of foodborne pathogens and *in vivo* colonization. This figure has been reproduced from ref. 44 with permission from Xia, *et al.* Copyright 2021. (D) Overview of the multiplexed CLAMP assay. This figure has been reproduced from ref. 45 with permission from Roh, *et al.* Copyright 2023.

Recent reports indicate that hydrogel microspheres can serve as containers for CRISPR/Cas reactions, with widespread applications in biosensing. The porous internal structure of hydrogel microspheres facilitates unhindered movement of biomolecules, resembling diffusion and reaction kinetics in solution. Yoon Ho Roh and colleagues leverage these advantages in CRISPR/Cas detection by integrating Cas into hydrogel microspheres, simplifying multiplex nucleic acid detection while preserving Cas enzymatic activity. Termed CLAMP (Cas-Loaded Annotated Micro-Particles), this CRISPR/Cas system based on HMPs (hydrogel microspheres) is designed for multiplex nucleic acid detection. Roh *et al.* immobilize Cas proteins within spatially encoded nanogel microspheres, where each HMP labeled with spatial codes targets unique nucleic acid sequences. Exploring its potential clinical applications, CLAMP is applied to detect high-risk human papillomavirus (HPV) DNA in cervical brush samples.^45^ Additionally, the realm of utilizing three-dimensional DNA nanostructures, encompassing DNA origami, DNA dendrimers, and DNA tetrahedrons in conjunction with CRISPR/Cas12 for biological detection and delivery, remains relatively underexplored. This domain holds immense potential for future advancements in sensor applications and various other scientific endeavors, suggesting promising prospects for development (Fig. 6).

### 4.3 The structure and mechanism of CRISPR/Cas13

**Scheme 5:**
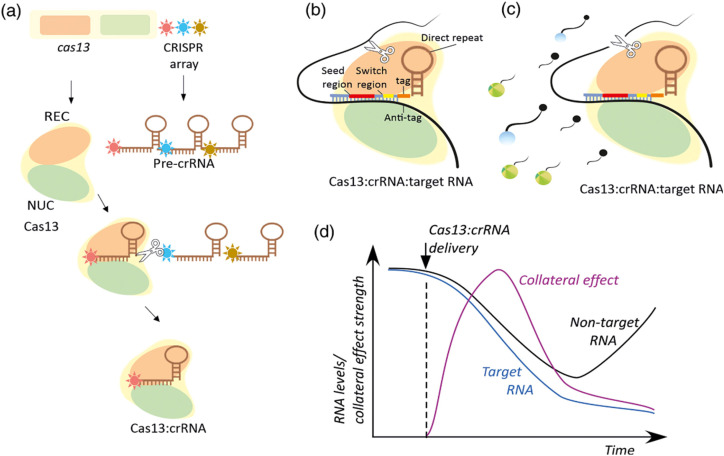
Molecular mechanisms of CRISPR-Cas13 expression and interference. (a) The expression of Cas13 and the CRISPR array produces the multifunctional Cas13 effector protein and the pre-crRNA that is processed into crRNA by Cas13s REC. (b) Base pairing between the spacer region of a crRNA and the single-stranded region of a target RNA in the context of the Cas13:crRNA complex sets the stage for target RNA cleavage by the Cas13 NUC lobe (c). Accessibility to the catalytic site leads to nonspecific degradation of bystander RNA. In part (c) the bystander RNA is an artificial reporter RNA used in SHERLOCK approaches described below. The reporter includes Cas13 nuclease-preferred squence, a 50 fluorescent dye, and a 30 quencher. Upon cleavage, a fluorescent signal is produced. (d) A hypothetical time-course of collateral effect, target RNA, and nontarget RNA levels upon induction of Cas13:crRNA expression. This scheme has been reproduced from ref. 108 with permission from Kordyś, *et al.* Copyright 2022.

CRISPR/Cas13 systems are RNA-targeting nucleases with high programmability and specificity. Research into their structure and mechanism has revealed various unique characteristics and potential applications. Guided by crRNA (CRISPR RNA), Cas13 nucleases primarily recognize and bind to target RNA segments, subsequently.^102^ This process involves crRNA binding to target RNA and conformational changes in the Cas13 protein, ultimately activating its nuclease activity.^103,104^ Structurally, the Cas13 protein family comprises multiple subtypes (*e.g.*, Cas13a, Cas13b, Cas13d), each with distinct structural features and functional mechanisms. For instance, Cas13bt3 is a compact variant activated in a length-dependent manner on target RNA and primarily cleaves at internal “UC” sites.^105,106^ Furthermore, structural studies of Cas13bt3 have revealed its conformation in fully activated states, guiding further engineering efforts.^106^ Cas13's self-defense mechanism is also a significant focus in its structural studies. For instance, through cryo-EM structural analysis, researchers have identified a mechanism that inhibits self-cleavage, achieved by complementary binding of tags and anti-tags to prevent degradation of its own RNA.^107^ In terms of applications, Cas13 is considered a promising RNA-targeting platform due to its lack of dependence on protospacer flanking sequences (PFS), ease of packaging, and non-permanent damage, making it suitable for various applications such as RNA knockdown and transcriptional regulation, particularly showing broad prospects in nucleic acid diagnostics and gene therapy.^102^ In summary, the CRISPR/Cas13 system achieves efficient RNA targeting and degradation through its unique structure and mechanisms, providing robust tools and platforms for both basic research and clinical applications.^106^ The CRISPR/Cas13 response mechanism is shown below (Scheme 5).^108^

#### 4.3.1 The application of CRISPR/Cas13 system combined with DNA nanotechnology

CRISPR/Cas13 has shown tremendous potential in cancer diagnosis, treatment, and research. Its RNA-targeting capability makes it a promising tool for early detection and monitoring of cancer biomarkers, without the need for complex instrumentation.^109^ CRISPR/Cas13 with DNA detection technology represents an emerging tool for both gene editing and diagnostics, characterized by high sensitivity and specificity. This technology utilizes the unique RNA-cleaving activity of Cas13 protein, guided by crRNA to target and cleave specific RNA sequences for detection. Additionally, Cas13's “collateral cleavage” activity allows it to cleave fluorescent reporter molecules upon recognizing target RNA, enabling detection of the target sequence's presence. The application of DNA nanotechnology enhances the safety and effectiveness of CRISPR/Cas systems in diagnosing and treating infectious diseases^110^^.^ This combined approach opens new avenues not only in gene editing but also in broader biomedical applications.

Researchers have developed a series of biosensing platforms for nucleic acids, proteins, small molecules, metal ions, *etc.*, utilizing the collateral cleavage activity of Cas12a, Cas14a, and Cas13a/b. Several notable examples of CRISPR-based biosensing systems include SHERLOCK, HOLMES, DETECTR, among others. In these biosensing systems, single-stranded DNA (or RNA) signal reporter probes are introduced into the CRISPR-Cas reaction system. By detecting changes in specific signals including fluorescence and colorimetric changes, precise monitoring of the specific recognition events conducted by Cas nucleases towards target DNA (or RNA) sequences is enabled. Nonetheless, a majority of these innovative CRISPR-based biosensing methodologies heavily depend on fluorescent reporter probes, necessitating the utilization of specialized optical detection equipment or instruments. Consequently, this reliance restricts their extensive application in point-of-care testing (POCT) scenarios.

Overall, research combining CRISPR/Cas13 with nanotechnology demonstrates significant potential in precise and efficient gene detection and therapy.

#### 4.3.2 CRISPR/Cas13 system combined with two-dimensional DNA nanostructures

CRISPR/Cas13 systems have wide applications in the biomedical field. The following section mainly discusses the application of CRISPR/Cas13 systems combined with 2D DNA nanostructures like SDA and RCA.

MicroRNAs (miRNAs), a class of small molecules with significant regulatory functions, have been widely used as biomarkers for early diagnosis of various diseases in the field of biosensing. Therefore, the development of a highly sensitive and specific miRNA detection platform is crucial. Liwen Guan *et al.* designed an enzyme-cyclic amplification system based on CRISPR/Cas13,^46^ using magnetic upconversion nanoparticles (MUCNPs) as biosensors to detect miRNA with high sensitivity and fidelity. Benefiting from the high fidelity and specificity of CRISPR/Cas13a, the triggering enzyme cyclic amplification, and the high fidelity and specificity of UCNPs within MUCNPs, this method demonstrates a high sensitivity and specificity in miRNA detection. Even in complex environments containing 10% fetal bovine serum (FBS) and serum samples, the method maintains high sensitivity and specificity in miRNA detection. Additionally, the detection limit of this method can be as low as 83.2 fM.

Based on CRISPR, cccDNA detection is an ultra-sensitive and highly specific method for detecting HBV cccDNA. Xiangying Zhang's team has developed a new strategy named CRISPR-based cccDNA detection method.^47^ The CRISPR-based cccDNA detection involves sample preprocessing, amplification, and detection steps. During the amplification step, the preprocessed sample serves as a template for RCA amplification *via* PCR to increase the target sequence's quantity. T7 RNA polymerase is used to transcribe the target sequence into single-stranded RNA (ssRNA). The T7 promoter sequence is attached to the front of the PCR product, enabling the PCR product to be transcribed into ssRNA with a specific target crRNA detected by Cas13a. In the detection step, specific crRNA guides Cas13a to recognize the target RNA complementary to crRNA intervals, triggering Cas13a-mediated reporter RNA cleavage for real-time detection of the target. CRISPR-based cccDNA detection is an ultra-sensitive and highly specific method for detecting HBV cccDNA. It provides a powerful tool for clinical treatment and effectively guides patients receiving long-term anti-HBV therapy to improve their therapeutic efficacy. Further research is needed to optimize the amplification and detection steps of this method and validate the connection between liver tissue and extrahepatic HBV cccDNA.

**Fig. 7:**
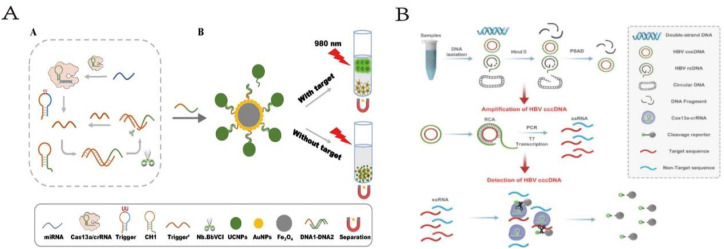
(A) Scheme of highly sensitive and specific miRNA detection method based on magnetic upconversion nanoparticle enhancement and CRISPR/Cas13a-driven signal amplification. This figure has been reproduced from ref. 46 with permission from Guan, *et al.* Copyright 2023. (B) Design and working principle of the CRISPR-based cccDNA assay for HBV cccDNA detection. This figure has been reproduced from ref. 47 with permission from Zhang, *et al.* Copyright 2022.

The application of CRISPR/Cas13 systems combined with three-dimensional DNA nanostructures is less explored in related fields, making it a promising direction for future development (Fig. 7).

## 5. Conclusion and prospects

DNA nanotechnology leveraging its unparalleled engineering prowess and biocompatibility, has garnered extensive application in the biomedical realm, showcasing vast potential across diverse disciplines.^111^ For several decades now, adhering to the fundamental Watson–Crick base pairing principles, DNA has been harnessed as a versatile programmable building block for constructing precisely molecularly defined nanostructures. The intricate size, shape, and configuration of these nanostructures can be meticulously tailored through strategic sequence design, facilitating the creation of DNA architectures with unparalleled versatility and arbitrary geometries. DNA nanotechnology has made significant strides in drug delivery and gene therapy. Through molecular recognition, DNA nanotechnology can build nanostructures with precisely controllable size, shape, and surface chemistry, which are crucial for drug delivery processes. Additionally, DNA nanotechnology has been instrumental in advancing biosensor technology, significantly boosting their performance metrics such as sensitivity, specificity, speed, and reproducibility through innovative interface engineering strategies.^112^ These sensors exhibit versatility in detecting a broad spectrum of biomolecules, ranging from nucleic acids and proteins to small molecules and ions pertinent to physiological processes.^113–115^ The application of DNA nanostructures in biomedical sciences spans multiple areas, including biosensing, bioimaging, drug delivery, and therapy.

The CRISPR/Cas system is a revolutionary genetic editing tool that has been widely applied in biomedical and biotechnological fields. Originally discovered in prokaryotes as part of their adaptive immune system, this system can defend against the invasion of foreign nucleic acids such as viral genomes and plasmids. Overall, the CRISPR/Cas system, as a powerful genetic editing tool, despite facing numerous challenges, holds immense potential in basic research, clinical applications, and biotechnology. Scientists are dedicated to overcoming these challenges to achieve its broader and safer application. The simplicity and efficiency of the CRISPR/Cas9 system have facilitated its widespread use in genome engineering, enabling editing of DNA not only in eukaryotic cells and organisms but also regulating gene expression.^116^ The extensive application of the CRISPR/Cas system has significantly advanced biological research, particularly through structural studies of the CRISPR/Cas12 variant. These studies have unveiled its immense potential as a versatile genetic editing and molecular detection tool within the realm of structural biology. Meanwhile, the CRISPR/Cas13 system, an RNA-targeting ribonuclease, exhibits high programmability and specificity. Research into its structure and mechanisms has revealed several unique characteristics and promising applications. The combination of CRISPR/Cas systems with DNA nanotechnology demonstrates enormous potential in the fields of gene editing and biomedicine. The integration of DNA nanotechnology with CRISPR/Cas systems has become the foundation for developing advanced pathogen detection technologies. This union not only enhances the efficiency and accuracy of gene editing but also lays the groundwork for achieving more complex functionalities in the future. DNA nanotechnology, owing to its inherent programmability, precise base pairing capabilities, and exceptional biocompatibility, emerges as an optimal vehicle for the delivery of CRISPR/Cas tools. Researchers have successfully constructed various DNA nanostructures, ranging from stationary branched DNA junctions and self-assembling nanostructures to intricate two-dimensional and three-dimensional constructs. These tailored structures furnish CRISPR/Cas systems with a highly customizable and modular platform, facilitating their integration and application The biorecognition detection methods discussed in this article, combining DNA nanotechnology with CRISPR/Cas systems, include HCA using two-dimensional DNA nanotechnology, CHA, and RCA. Three-dimensional DNA nanotechnology involves DNA tetrahedrons, G-quadruplexes, DNA hydrogels, *etc.* These technologies empower biosensors with the capability to detect minute entities like DNA, RNA, and microorganisms. Consequently, the synergistic integration of these advancements not merely boosts the efficacy of CRISPR/Cas systems but also forges novel paths for addressing both established and unknown genetic disorders. DNA nanotechnology has expansive application prospects within biomedical science, pioneering various new biomedical applications through its unique programmability, functionalization, and dynamic responsiveness. However, DNA nanotechnology still faces several challenges in clinical translation, including stability *in vivo*, immune responses, and large-scale production. Information on further research and addressing these challenges will help propel the practical application of DNA nanotechnology in biomedicine. The combined application of CRISPR/Cas systems with DNA nanotechnology has shown unprecedented potential in gene editing and biomedical fields, particularly in enhancing the delivery efficiency and functional diversity of CRISPR/Cas systems. DNA nanotechnology and CRISPR/Cas system can realize the detection of various biomarkers at the laboratory level, which has a broad application prospect in the fields of biosensing, medical diagnosis and biopharmaceuticals. Despite the impressive performance of CRISPR/Cas systems in laboratory research, their clinical application still encounters various obstacles. These problems, which urgently need to be researched and solved, are mainly (1) the limitations of nucleic acids themselves. Nucleic acid aptamers used for biomarker detection have cross-reactivity between them, and the detection process of nucleic acid is complex, harsh conditions, different laboratories for the same target detection of nucleic acid affinity, specificity, and other chemical parameters such as differences, which leads to nucleic acid in the diagnosis of human diseases can not be accurately identified, it is believed that more stringent screening conditions can be designed to have the optimal conditions of the reaction system. It is believed that through more stringent screening conditions, the reaction system with optimal conditions can be designed to realize the accurate recognition of biomarkers for clinical use in the diagnosis and treatment of diseases. (2) High dependence of detection environment. Although the fluorescent, colorimetric and electrochemical biosensors based on DNA nanotechnology combined with the CRISPR/Cas system can greatly improve the detection performance of traditional biosensors, the nucleic acid aptamer and the CRISPR-Cas system are less adaptable to the detection environment, and the matrix effect has a greater impact, which leads to its reliability and reproducibility are not high in complex practical applications, and even poor detection results. Therefore, it is necessary to further explore the detection of DNA-based nanotechnology combined with CRISPR/Cas system in the practical application environment, and to screen biocompatible and stable nanomaterials to be added into the system to improve the detection performance of the target in the complex environment. (3) The scope of application is limited. Although these proposed methods have already realized the detection of various biomarkers from small molecules and proteins to bacteria, viral particles and tumor cells, it has not yet been able to realize the instantaneous detection (POCT), so in the future, researchers can introduce portable devices based on biochips and microfluidic devices into this method to realize the instantaneous detection of biomarkers, and expand the application of DNA nanotechnology combined with the CRISPR/Cas system in biomedical engineering. In the future, portable devices based on biochips and microfluidic devices can be introduced to realize instant detection of biomarkers, thus expanding the application of DNA nanotechnology combined with CRISPR/Cas system in biomedical engineering.

Overall, DNA nanotechnology holds immense potential in enhancing the delivery efficiency and specificity of CRISPR/Cas systems, yet significant hurdles persist in refining delivery vehicles, enhancing targeting specificity, and advancing their clinical utilization. Overcoming these challenges is paramount to realizing the full potential of this groundbreaking technology.

## Data availability

No primary research results, software or code have been included and no new data were generated or analysed as part of this review.

## Author contributions

YH drew schematic diagram and wrote the manuscript. ZC and HH revised the manuscript. SD provided the overall framework design of the review. ZM provided funding. All authors read and approved the final manuscript.

## Conflicts of interest

The authors declare that they have no known competing financial interests or personal relationships that could have appeared to influence the work reported in this paper.

## Supplementary Material

RA-015-D4RA08325C-s001
