# Supplementary material for: Important applications of DNA nanotechnology combined with CRISPR/Cas systems in biotechnology
Source: RSC Adv. 2025 Feb 25;15(8):6208–30. doi: 10.1039/d4ra08325c (PMC11851101; doi:10.1039/d4ra08325c)
Supplement: RA-015-D4RA08325C-s001 [file RA-015-D4RA08325C-s001.pdf]

Supplementary Information (SI) for RSC Advances.
This journal is © The Royal Society of Chemistry 2025

Data Available statement: Data sharing is not applicable to this article as no new data were created or analyzed in this study.
